# An Analysis of Arguments Advanced via Twitter in an Advocacy Campaign to Promote Electronic Nicotine Delivery Systems

**DOI:** 10.1093/ntr/ntac237

**Published:** 2022-10-21

**Authors:** Ell Lee, Janet Hoek, Elizabeth Fenton, Ayush Joshi, Karen Evans-Reeves, Lindsay Robertson

**Affiliations:** Otago Medical School, University of Otago, Dunedin, New Zealand; Department of Public Health, University of Otago, Wellington, New Zealand; Centre for Bioethics, University of Otago, Dunedin, New Zealand; Tobacco Control Research Group, Department for Health, University of Bath, UK; Tobacco Control Research Group, Department for Health, University of Bath, UK; Tobacco Control Research Group, Department for Health, University of Bath, UK; Department of Preventive and Social Medicine, University of Otago, Dunedin, New Zealand

## Abstract

**Introduction:**

Advocates of electronic nicotine delivery systems (ENDS) increasingly use Twitter to promote liberal ENDS policies. “World Vape Day” (WVD) is an annual campaign organized by pro-ENDS advocacy groups, some of which have links to the nicotine industry (eg, via funding from the “Foundation for a Smoke-Free World”). In 2020, the campaign used dedicated social media accounts to disseminate WVD-branded images and campaign messages. We examined tweets posted as part of WVD 2020 to identify and analyze pro-ENDS policy arguments.

**Aims and Methods:**

We extracted tweets posted between 26 May and 3 June 2020 that included the hashtag #WorldVapeDay. We used qualitative thematic analysis to code a random sample (*n* = 2200) of approximately half the original English language tweets (*n* = 4387) and used descriptive analysis to identify the most frequently used co-hashtags.

**Results:**

Arguments related to four themes: *harm reduction, smoking cessation, rights and justice, and opposition to ENDS restrictions*. Tweets criticized individuals and groups perceived as opposing liberal ENDS regulation, and used personal testimonials to frame ENDS as a harm reduction tool and life-saving smoking cessation aid. Tweets also advanced rights-based arguments, such as privileging adults’ rights over children’s rights, and calling for greater recognition of consumers’ voices. Tweets frequently used hashtags associated with the WHO and World No Tobacco Day (WNTD).

**Conclusions:**

The WVD campaign presented a series of linked pro-ENDS arguments seemingly aimed at policy-makers, and strategically integrated with the WHO’s WNTD campaign. Critically assessing pro-ENDS arguments and the campaigns used to promote these is vital to helping policy actors develop proportionate ENDS policy.

**Implications:**

Social media platforms have considerable potential to influence policy actors. Tweets are easily generated and duplicated, creating an impression of sizeable and influential stakeholders. Evidence that the “World Vape Day” campaign was supported by groups with industry links, and targeted—at least in part—at WHO officials and those who follow the WHO World No Tobacco Day campaign, highlights the importance of critically reviewing such campaigns. Further research could examine how health advocates could engage in pro-ENDS campaigns to support balanced messaging and informed policy-making.

## Introduction

Several studies conclude that electronic nicotine delivery systems (ENDS) are less harmful than smoking^1, 2^; however, whether and how ENDS could contribute to public health goals remains uncertain. Disagreements continue over their long-term risks,^2^ the effect of second-hand ENDS aerosol,^3^ and whether ENDS support or inhibit smoking cessation.^4–6^ Systematic reviews published in 2021 indicated ENDS may be marginally more effective for smoking cessation than nicotine-replacement therapy, yet the data included in those reviews do not suggest they are a panacea that will end the smoking pandemic.^7, 8^ For instance, one concluded that for every 100 people using nicotine-containing ENDS to stop smoking, approximately 9–14 people would stop successfully, compared with approximately 6 of 100 people using nicotine-replacement therapy.^8^ Furthermore, significant concerns persist over ENDS’ appeal to youth^9, 10^ and potential “gateway” role to tobacco smoking.^11^ Patterns of youth and young adult ENDS use vary across countries. For instance, in the United States, around 20% of high-school students and 5% of middle-school students were current ENDS users in 2020,^12^ and the prevalence of ENDS use among those groups appears to be declining. In contrast, in many other countries including Australia and New Zealand, youth and young adult ENDS use is increasing and is highest among those aged 18–24 years old; in 2019, 5% of Australian 18–24 year olds were current ENDS users (defined as at least monthly usage),^13^ compared to 21% in New Zealand.^14^ Differences in ENDS use across countries reflects policy variations in how ENDS are regulated,^15, 16^ and debate continues across scientific, advocacy and policy communities about the best regulatory approach.^17^ This regulatory divergence creates incentives for stakeholders and interest groups to influence policy using the media, including social media, to present an issue, communicate arguments and potential solutions, and mobilize support.^18^

Despite ongoing debates about ENDS’ potential risks and benefits, and impact on smoking cessation, several studies report that the “Twittersphere” of ENDS-related information is dominated by endorsements of ENDS as effective smoking cessation aids.^19–24^ For example, a 2015 study of ENDS-related Twitter conversations concluded that pro-ENDS narratives shaped discussions about ENDS bans or taxation.^25^ Tweets questioned scientific evidence of ENDS’ health risks, argued ENDS regulations would have unintended consequences, and presented ENDS as life-saving smoking cessation devices.^25^ Some research identified coordinated efforts by ENDS proponents, including “Twitter bombing” to oppose regulation,^26^ and counter-campaigns that parodied public health initiatives.^27, 28^

On March 22, 2012, ENDS advocacy organizations, including Consumer Advocates for Smoke-Free Alternatives Association (CASAA), initiated the first “World Vaping Day” (as it was originally called before being renamed “World Vape Day”).^29^ While many national and regional ENDS advocacy campaigns exist, WVD has become a significant global advocacy platform for ENDS proponents, who have used it to try to influence the World Health Organization (WHO). For instance, CASAA’s 2014 WVD “International Call to Action” urged ENDS consumers to contact the WHO to oppose bans on ENDS use in public places and present evidence suggesting exposure to second-hand ENDS emissions is not harmful.^30^ The recent rescheduling of WVD, which moved to 30 May in 2020, one day prior to the WHO’s World No Tobacco Day, arguably attempted to integrate pro-ENDS messages into the WHO’s anti-tobacco campaign. The International Network of Nicotine Consumer Organisations (INNCO), an umbrella organization for national and regional ENDS consumer advocacy groups that has received funding from the Philip Morris International-funded Foundation for a Smoke-Free World (FSFW),^31, 32^ managed the 2020 WVD website. The World Vapers’ Alliance, another key organization involved in WVD, is also reportedly supported by tobacco industry funding.^33, 34^ The campaign used specially created WVD-branded images and messages, and disseminated these via Twitter (@worldvapeday) as well as Facebook and Instagram (see: www.facebook.com/WorldVapeDay and https://instagram.com/world_vape_day). The WVD website encouraged ENDS users to participate by posting testimonials and stated that: “Our goal is to present an assortment of compelling individual reports that will make it impossible for anyone to claim that substitution of low-risk alternatives is not a proven method for quitting smoking”.^35^

Evidence WVD has become a more organized global advocacy campaign highlights the need to examine how its messages are used to influence policy, given arguments made in these are likely to be repeated across many jurisdictions, regardless of differences in policy context. We focused on Twitter data, since Twitter has provided a popular platform among policy advocates, and earlier analyses of Twitter data have provided valuable insights into policy debates.^31, 36, 37^ We analyzed tweets posted as part of WVD 2020 to identify and critically review the arguments presented.

## Methods

### Data Collection

Using automated scraping, we downloaded a dataset of tweets that included the hashtag #WorldVapeDay. We collected 58 551 tweets posted over eight days from May 26 to June 3, 2020 (World Vape Day was on May 30, 2020). After excluding non-English language tweets, the dataset comprised 39 471 tweets (4387 original tweets and 35 084 retweets) from 2289 unique users. We assigned each original tweet an identifier and used an online random number generator (www.numbergenerator.org) to draw a random sample of just over half of the original English language tweets (*n* = 2200) for content coding. We used NVivo12 to manage the content coding.

### Coding and Analysis

LR and JH reviewed the Twitter data and developed a draft codebook to classify tweets that put forward an argument, position, or call to action in relation to ENDS. Our primary analysis used qualitative thematic analysis and emergent (rather than a priori) coding to develop codes from the tweets^38, 39^; we coded concurrently and iteratively for a supplementary content analysis, where we compared the relative frequency of the different arguments. LR and JH tested the initial codebook with a random sample of *n* = 200 tweets; EL coded the same *n* = 200 tweets as a training exercise before LR reviewed the coding and refined the categories and coding processes. LR and EL next independently coded a second random subset of *n* = 200 tweets and assessed inter-rater reliability using Cohen’s Kappa. Arguments with a minimum of 20 coded tweets (*n* = 17) were assessed (as the effect of bias is greater with a smaller number of comparisons),^40^ with a resulting average Kappa = 0.68. LR and EL discussed and resolved coding discrepancies (re-coding where necessary and as mutually agreed) and further refined definitions and delineations. A third random subset of *n* = 200 tweets was coded using the same process; Cohen’s Kappa increased to 0.77 and the percentage agreement averaged 96.3%. EL randomized and coded the remaining tweets. LR, EL, EF, and JH met regularly to review the coding, and discuss, compare and agree on classifications. After coding all arguments and positions, LR, EL, EF, and JH met and developed the four over-arching themes these supported: *Harm reduction, smoking cessation, rights and justice, and opposition to ENDS restrictions*. Supplementary File 1 contains the final codebook and illustrative tweets.

Tweets that did not advance an argument, position, or call to action in relation to ENDS, or that contained insufficient or incomprehensible text, were coded as “no argument” and excluded. We did not include tweet hashtags in our qualitative analysis unless they were integrated into the core message of a tweet and thus helped communicate the argument, position or call to action. However, we undertook a supplementary descriptive analysis to identify the most common hashtags. Supplementary File 2 provides an overview of the analytical process.

### Ethical Considerations

A delegated authority acting on behalf of the Head of the Department of Public Health (University of Otago Wellington) reviewed and approved the study as a low-risk project (D21/047). The study also received ethical approval from the REACH Committee at the University of Bath (ref: EP17/18 237) and complies with Twitter’s terms of service.^41^

## Results

Table 1 shows the most prevalent arguments relating to each of the four themes we identified. Arguments with fewer than 10 coded tweets are shown in Supplementary File 3. Because tweets including only a “call to action” differed qualitatively from those containing an argument or position, we present the number of coded “call to action” tweets in Supplementary File 3. Any tweets containing *both* a “call to action” *and* an argument are included in Table 1.

**Table 1.** Definition and Frequency of Arguments about ENDS, by Theme

THEME; *Argument*	Definition	Frequency*	Proportion^
HARM REDUCTION			
*Harm reduction*	Makes a general harm reduction claim(s) without specific 95% claim; frames ENDS as low-risk, lower risk, or a safer alternative.	350	22%
*At least 95% less harmful*	Includes a reference to the “ENDS are 95% safer than smoking” claim (or cites a higher %, such as “99% safer”).	120	8%
*Nicotine not harmful*	States that nicotine itself is not the harmful constituent of cigarettes.	76	5%
*Improved health outcomes*	Claims switching to ENDS from smoking leads to improved health outcomes (i.e., generally rather than personal experience).	71	4%
*Vaping is not smoking*	Makes argument or statement about how vaping is not smoking.	34	2%
*secondhand vape not harmful*	States that secondhand aerosol produced by ENDS is benign or harmless.	17	1%
**SMOKING CESSATION**			
*Testimonial*	Promotes ENDS use using a testimonial from a former cigarette smoker.	272	17%
*Improved symptoms*	Refers to tweeter’s own improved health/ symptoms, which they attribute to ENDS (i.e. subset of testimonials).	*32*	*2%*
*ENDS save lives*	Describes ENDS as life-saving or as having saved the tweeter’s own life	239	15%
*ENDS support quitting*	Refers to or frames ENDS as a smoking cessation or “quitting” aid (excludes testimonials).	79	5%
*Effects of smoking*	Refers to smoking-related harm either at population level or effects on loved ones.	64	4%
*ENDS’ superiority*	States ENDS are more effective/ better than other cessation methods.	56	4%
*Cessation success*	Cites ENDS-led cessation rates.	13	1%
**RIGHTS AND JUSTICE**			
*Access to flavors*	Refers favorably to flavored ENDS (i.e. importance of flavored ENDS in stopping smoking).	169	11%
*Right/ freedom to choose*	Argues smokers’ access to ENDS and/ or harm reduction is a human right; smokers/ adults) should have freedom of choice.	166	10%
*Vaping is for adult smokers*	Frames ENDS as targeted at and/ or used by adults and/or smokers only; not for children or youth.	114	7%
*Industry is consumer-driven*	Frames ENDS as developed and made by smokers for smokers; ENDS industry as consumer-driven/ grassroots.	44	3%
*Involve consumers*	Argues policy-makers/ regulators must listen to, learn from and/ or involve ENDS consumers in ENDS policy-making.	26	2%
*Don’t deserve to die*	Argues nicotine users “do not deserve to die”.	21	1%
*Refutes “gateway” hypothesis*	Refutes the idea or evidence that vaping can lead to smoking.	17	1%
*Right to know the truth*	Argues public have the “right to know the truth” about ENDS, or have a right to be fully informed.	10	1%
*Youth ENDS trial inevitable*	Argues cannot prevent all youth from ENDS experimentation and/ or refutes concern over youth ENDS trial or usage.	10	1%
**OPPOSITION TO ENDS RESTRICTIONS**			
*Criticism of those perceived as opposing ENDS*	Criticizes individuals or organisations perceived as opposing ENDS; tweet may include “Quit or Die” mantra in reference to tobacco control efforts.	448	28%
*Misinformation about ENDS*	Argues or implies “misinformation” and/ or selective evidence used about ENDS; calls for “evidence”; implies ENDS advocates disseminate factual information.	117	7%
*Repeal ENDS ban*	Appeals for an existing ban on ENDS to be removed e.g. in India or Australia.	69	4%
*Restrictions unjustified*	Frames restrictions on ENDS as prohibition; argues restrictions are unnecessary, unjustifiable, infeasible or ineffective.	64	4%
*Unintended consequences*	Refers to possible unintended consequences of policies such as flavor bans, or a ban on ENDS.	25	2%
*International comparisons*	Refers to countries with desirable ENDS policies e.g. NZ or UK, or to those with undesirable policies e.g. Australia or India.	13	1%

N.B. *Total coded tweets (*n* = 1590; ie excluded *n* = 610 tweets containing no argument). ^Proportion indicates the % of *n* = 1590 tweets that included the argument; tweets were not mutually exclusive. Our definition of “argument” is expanded to include types of evidence (ie, Testimonials). Coding of arguments with fewer than 10 tweets is shown in Supplementary File 3, as is coding of “Call to Action” tweets (any specific arguments conveyed in the latter group of tweets are included in the table above).

### Thematic Analysis

#### Harm Reduction

Tweets in the harm reduction theme presented ENDS as a less harmful, safer, or “*healthier*” alternative to smoking. Some comments compared ENDS to other risk reduction strategies: “*Motorcycle helmets, condoms, masks, shoes, clothing, hard hats, sunscreen, seatbelts, sunglasses, ear plugs. We all practice [sic] harm reduction literally every day . . .”.* Several cited scientific references: (eg, “*British Medical Association: There are clear potential benefits to #ecig use in reducing the substantial harms associated with smoking, and a growing consensus that they are significantly less harmful than tobacco use”).* Others referred to Public Health England’s conclusion that ENDS are **95% less harmful** than smoking (eg, *“We already know #vaping is at least 95% safer than smoking!”*). Tweets substantiated harm reduction arguments using claims about **improved health outcomes** (eg, *“Switch to vaping and improve your heart and lung health”*).

Several tweets argued that **nicotine is not harmful** and implied ENDS avoided the serious risks caused by cigarette constituents (eg, “*. . . people smoke for the #nicotine but die from the TAR.”,* and *“how many people die from #nicotinevaping? It’s an easy one, zero”*). Tweeters argued that **vaping is not smoking** by claiming that ENDS do not combust or produce smoke, hence could not be smoking: “*. . . Something must at least contain smoke to be rightfully called smoking. Where there’s no smoke, there’s no one smoking.”* Seventeen tweets asserted that **second-hand ENDS aerosol was not harmful** and framed emissions as having either “minimal” or no toxic effects (eg, *“..there are no proven health risks of second hand vaping to the health of bystanders. Vaping could prevent millions of deaths from secondhand smoke”*). Six tweets (see Supplementary File 3) diminished ENDS’ potential risks by comparing these to daily life activities: “. . . *Relative risk matters, and relative to smoking, drinking, and other risky behaviors people engage in, vaping nicotine is pretty benign”.*

#### Smoking Cessation

Most tweets in this theme used personal ***testimonials*** to present ENDS as an effective smoking cessation aid. Comments typically outlined how long individuals had smoked, when they had switched to using ENDS, the e-liquid flavor used, and the ease of quitting by using ENDS (eg, *“I’m a survivor. I stopped smoking with pistachio cream and peanut-waffle flavored e-liquids. Nonsmoker since 2018. Started smoking when I was 12 years old, smoked for 47 years”*). Testimonials frequently claimed that **ENDS save lives**, **support quitting,** and are **superior** to other smoking cessation methods, such as nicotine replacement therapy (NRT): *“I’m 60 years old and smoked for over 37 years. Tried every Pharma and NRT product to quit -- they all failed. If it wasn’t for E-liquid Vape Products getting me off cigarettes, I would still be smoking (or I would be dead from smoking)*”. Many corroborated these arguments by outlining **improvements in symptoms** experienced when smoking: “*. . . almost 5 years smoke free, and feeling and breathing a lot better than before”,* or by citing ***cessation rates*** to highlight ENDS’ superiority over other cessation approaches: *“. . . [vaping] has an amazing 18% cessation success rate”.*

Personal stories also outlined the **effects of smoking** on loved ones and ENDS’ potential life-saving effects for individuals (eg, “*I wish vaping would’ve been around to save my dad. My dad died when I was 14 of emphysema due to horrible combustible cigarettes”)* and the population (eg, “*Cigarettes kill 480,000 Americans a year!*”). While nearly all tweets in this theme hailed ENDS as an effective cessation aid, few referred to dual use (ie, concurrent smoking and ENDS use), which may impede smoking cessation (see Supplementary File 3).

#### Rights and Justice

Tweets in this theme asserted users’ **freedom to choose** ENDS, and framed access to ENDS as a human right and civil liberty (eg, *“It’s your right to choose a less harmful product!”*). Tweets encouraged others to “fight for their rights”: *“Everyone has the right to quit smoking combustible cigarettes. Everyone should have the right to use vaping to do that. I will always fight for people to be able to quit smoking like I did”.* Numerous tweets represented nicotine users who did not or could not quit smoking as having been abandoned without cessation options (“. . . *people****don’t deserve to die****just because they use nicotine”).* Tweeters implied authorities misled the public about ENDS and claimed people had the **right to know the truth** that ENDS were effective cessation aids (eg, *“The American people have a right to know the truth about flavored nicotine e-liquid vaping; 0 deaths 18% cessation, 80% smoker transition, 95% less harmful.”*)

Rights-based arguments frequently claimed that ENDS **flavors** supported smoking cessation (eg, *“Quitting smoking was made possible for me and millions of others by way of flavored vapes”*) and highlighted the importance of flavors to adult former smokers: *“I am 34 years old and flavored vapor products helped me quit a 15 year habit. Adults like flavors!”*. Presenting ENDS as an **adult product** implicitly rejected flavors’ appeal to young people and privileged the potential benefits adult smokers could gain from switching to ENDS above the potential risks nonsmoking youth would face from ENDS uptake: *“Politicians are using the kids as a shield but turn a blind eye and deaf ear to adults that have quit deadly combustible cigarettes. It’s time to listen and see that vaping isn’t trying to hook kids. It’s about savings adults’ lives”* and *“I am an adult. I have the right to make a less harmful choice. Please do not take away this right from me. Bring strong laws to control youth vaping, but don’t punish.. Indian smokers.”* Several tweets claimed that youth trial of ENDS was **inevitable,** though minimized the population health risk involved or **refuted the gateway hypothesis** (e.g., *“Youth are experimenting with vaping but experimentation alone is not a serious public health problem. Importantly, regular vaping among young people is rare and confined almost entirely to those who already smoke”)*. Tweeters thus argued that youth ENDS use did not justify restrictions they thought would impinge on adults’ rights (eg, *“Taking rights away from adults doesn’t protect kids”*) and held parents and adolescents themselves, not the ENDS industry, responsible for youth vaping. This reasoning enabled them to reject ENDS restrictions: (“*Parents, parent your kids and leave adult choices available for smokers!”* and *“I am an adult and I love flavored vapor products. Law abiding adults shouldn’t be punished for adolescent criminals”*).

Tweets also appealed to regulators to **involve consumers** in policy-making and recognize them as principal stakeholders. While some tweets adopted a defiant tone *(“We are #vapersvoice and we will not be #silent”),* others presented ENDS users as experts who embodied ENDS’ effectiveness as a smoking cessation aid (“*Data is evidence. The evidence is real. Check out more of the thousands [of] success stories submitted by REAL people . . .*”). Framing ENDS as a **consumer-driven,** grassroots industry authenticated these arguments and sharply differentiated ENDS users from tobacco companies: *“Vaping is a miraculous phenomenon on smoking cessation and public health exclusively led by consumers: we are not Big Tobacco!”*.

#### Opposition to ENDS Restrictions

Tweets in this theme challenged groups seen as unsupportive of ENDS and disparaged restrictive ENDS regulation. The most prevalent tweets **criticized individuals or organizations perceived as opposing ENDS;** for example, some tweets accused public health groups of having a vested interest in sustaining smoking to avoid jeopardizing tobacco control funding (eg, *“The ones that say no to THR are the ones profiting and collecting blood money from smokers. They lie about vaping so that you will hate the idea too”*). Many tweets accused the WHO of immorality (eg, *“We do know that the @WHO is for sale and has been bought out by the tobacco economy and now markets to children, however. Happy #WorldVapeDay you lying bastards*”). Tweets contrasted public health groups’ alleged spread of **misinformation** (eg, *“They are using media to create panic a ‘youth epidemic’ and spread misinformation to continue to profit from the established tobacco/pharma revenue stream!”)* with ENDS advocates’ dissemination of factual information: *“thank you to the #vaping community for so much passion, fortitude & most of all FACTS that were shared this weekend*”.

Many tweets presented **ENDS restrictions as unjustified** and argued that nicotine abstinence is not realistic: *“Bans don’t work, vape bans especially not, since nicotine is freely available in many other forms. Why ban the least harmful of them?”*. These claims supported calls to **repeal** restrictions (eg, *“Vaping, it saved my life, it’s the best NRT. We have to #EndVapeBan in India*”) and warnings of adverse **unintended consequences**: *“Bans/taxes just force adults/youth back to smoking that kills 1,300/day”.* Tweets used **international comparisons** to illustrate risks and benefits of different regulatory approaches: “*Several countries which banned e-cigarettes including Mexico, Brazil and Thailand saw a booming black-market - making it difficult for government to regulate the sales of these products”*) and *“The UK is a great example of a country encouraging smokers to switch. The switching rate is much higher than in other countries because they have embraced it”*. While many tweets criticized **individuals or organizations perceived as opposing ENDS** (*n* = 448), very few (*n* = 9) criticized the nicotine or tobacco industries (see Supplementary File 3).

#### Tweet content: Hashtags

Table 2 shows the ten most frequent hashtags, other than #WorldVapeDay. The most common, #SayYesToTHR, resembles an overt political message aimed at policy-makers, and several other frequently used hashtags reference either the WHO, World No Tobacco Day (WNTD), or the official WNTD2020 hashtag: #TobaccoExposed (indicated in bold text in Table 2). In contrast, hashtags containing more descriptive language, such as #harm reduction, #THR and #tobaccoharmreduction were far less prevalent (2%, 2% and 1%, respectively; not shown in Table 2).

**Table 2.** Ten Most Commonly used Hashtags in the 2020 World Vape Day Campaign

Hashtag	Frequency	%*
#SayYesToTHR	1440	33
#vaping	1035	24
**#TobaccoExposed**	**993**	**23**
**#WNTD or #WNTD2020**	**954**	**22**
#EndVapeBan	703	16
#VapingSavesLives	637	15
**#WorldNoTobaccoDay**	**464**	**11**
#vape	285	6
**#WHO**	**258**	**6**
#WeVapeWeVote	234	5

Denominator is the *N* = 4387 original tweets; THR = tobacco harm reduction; WNTD = World No Tobacco Day, the World Health Organisation (WHO) annual anti-tobacco campaign.

## Discussion

We identified four main themes in the tweets tagging the 2020 World Vape Day Twitter campaign: *harm reduction, smoking cessation, rights and justice,* and *opposition to ENDS restrictions*; those themes, and the specific arguments we identified, reflected campaign memes and messages disseminated by WVD organizers (eg, http://www.facebook.com/WorldVapeDay and https://instagram.com/world_vape_day). The most prevalent messages criticized those perceived as opposing liberal ENDS regulation, such as the WHO and other public health agencies, and represented them as immorally spreading disinformation about ENDS to maintain (rather than reduce) smoking prevalence. Widespread use of hashtags associated with the WHO enabled WVD tweets to “piggyback” on the trending topic of World No Tobacco Day and increase WVD messages’ reach.^20^ In contrast to previous studies,^19, 42^ we found very few overtly commercial ENDS messages (ie, those advertising specific ENDS products), which may reflect the evolution of WVD into a political campaign (rather than a more general absence of commercial ENDS tweets on Twitter). Instead, WVD messages promoted ENDS and “harm reduction” more generally, and the campaign appeared largely directed at regulators. WVD organizers encouraged personal testimonials that presented ENDS as a life-saving smoking cessation aid, in the hope of changing the attitudes of “those who want to restrict access to e-cigarettes and other smoke-free products”.^35^

ENDS consumer groups with industry links were involved in organizing WVD, including CASAA (founded and governed by ENDS industry representatives as well as consumers,^29, 43^) and INNCO (a Foundation for a Smoke-Free World grantee and thus an indirect recipient of PMI funding).^32^ Another organization central to WVD 2020, the World Vapers’ Alliance, which hosted a global online YouTube event as part of the campaign,^44^ has subsequently been the subject of investigations into its funding sources.^45^ One investigation claimed British American Tobacco has played “a central and hands-on role in orchestrating, directing and funding” the World Vapers’ Alliance and its social media activities.^34^

The links between key organizations involved in World Vape Day and the ENDS industry have important implications. The tobacco industry’s long history of creating and using third parties (including consumer groups) to disseminate its arguments,^46^ along with allegations of BAT’s involvement in the World Vapers’ Alliance,^34^ gives rise to the possibility that industry actors shaped the pro-ENDS messages disseminated during WVD. Direct industry involvement would raise questions about the ostensible grassroots nature of the WVD campaign and thus the validity of some of the arguments advanced. While it is beyond our study to conduct a full critical analysis of the WVD arguments, other research has outlined limitations in the industry’s “harm reduction” arguments,^47, 48^ and criticized ENDS proponents’ use of rights-based arguments that privilege adult smokers’ rights over children’s.^49^ Furthermore, although messages endorsing ENDS as effective smoking cessation aids dominate the “Twittersphere” of ENDS-related information,^19–25^ ENDS’ effectiveness as smoking cessation tools remains disputed, given a lack of rigorous randomized controlled trials.^7^ Lastly, although WVD appeared to target ENDS policy actors,^35^ the promotion of personal testimonials could represent a form of viral marketing promoting ENDS to nonusers. While Twitter has a policy banning tobacco advertising that applies to “alternatives which imitate the act of smoking” and “events sponsored by tobacco manufacturers”,^50^ it is unclear how that policy affects campaigns such as WVD, where industry links are neither declared nor apparent.

How could public health advocates address pro-ENDS campaigns such as WVD? Advocates could become more proactive in social media debates around ENDS; doing so could foster a clearer balance between ENDS proponents and health perspectives,^19, 51^ given health professionals typically do not engage in social media discussions.^24, 52^ While research suggests public health social media campaigns can change individuals’ attitudes, few studies have examined whether public health advocates’ real-time responses that aim to balance policy discussions are effective. Further research could examine how health advocates might engage in policy campaigns to support balanced messaging and informed policy-making. While social media platforms are increasingly used to influence policy actors,^53^ using Twitter to promote pro-ENDS messages is likely part of a wider strategy to influence ENDS policy, and the arguments we identified will likely appear in other outputs and lobbying activities. Our findings can thus inform possible responses by advocates, such as counter arguments where appropriate, using more varied platforms than Twitter alone. Further research could examine the extent to which WVD arguments have been adopted in policy-making proposals and wider stakeholders’ submissions on potential policy. Ongoing analysis of relationships between pro-ENDS campaigns and ostensibly independent entities that promote liberal regulation would help identify potential conflicts of interest that may not be immediately apparent, and would support compliance with FCTC Article 5.3.^54^ Lastly, public health advocates could continue advocating for greater regulation of social media to reduce all forms of nicotine and tobacco product promotions.^55^

Our study has several limitations. Our dataset may not have captured every tweet containing #WorldVapeDay and we did not include tweets from private Twitter accounts as we used Twitter’s public API. We analyzed a random sample of half, rather than all, the original tweets in our dataset, though random selection of the analyzed tweets means these are unlikely to differ systematically from those we did not analyze. The frequency table (Table 1) presents the most frequent themes and arguments, rather than outlining a precise estimate of the tweets conveying a particular argument. However, our use of manual coding, and the intensive consultation and review process used during the analysis, are important strengths that enabled the detailed qualitative examination required by our research question, and identified the prominence of rights-based arguments.

Policy-makers need to consider diverse arguments for and against liberalizing ENDS. Analyzing Twitter activity associated with WVD and the core arguments advanced has identified potential conflicts of interest, including roles played by industry-linked groups such as CASAA, INNCO and the World Vapers’ Alliance. By highlighting efforts to embed pro-ENDS messages into the WHO’s WNTD campaign, our analyses support a more informed and dispassionate assessment of ENDS advocates’ most common arguments. Tobacco control researchers and advocates should continue to monitor and report on obfuscated relationships between the nicotine industry and ENDS advocacy groups.

## Supplementary Material

A Contributorship Form detailing each author’s specific involvement with this content, as well as any supplementary data, are available online at https://academic.oup.com/ntr.

ntac237_suppl_Supplementary_Material_S1

ntac237_suppl_Supplementary_Material_S2

ntac237_suppl_Supplementary_Material_S3

ntac237_suppl_Supplementary_Taxonomy-form

## Data Availability

The data underlying this article will be shared on reasonable request to the corresponding author.

## References

[1] McNeill, A., Brose, L., Calder, R., Hitchman, S., Hajek, P., McRobbie, H. E-cigarettes: an evidence update. A report commissioned by Public Health England. 2015. www.gov.uk//government/uploads/system/uploads/attachment_data/file/457102/Ecigarettes_an_evidence_update_A_report_commissioned_by_Public_Health_England_FINAL.pdf. Accessed March 12, 2020 10.1136/bmj.h5010 26403170

[2] U.S. National Academies of Science Engineering and Medicine. Public Health Consequences of E-Cigarettes. 2018 http://nationalacademies.org/hmd/Reports/2018/public-health-consequences-of-e-cigarettes.aspx. Accessed March 12, 2020

[3] Fernández E , Ballbè M, Sureda X, et al. Particulate matter from electronic cigarettes and conventional cigarettes: a systematic review and observational study. Curr. Environ Health. 2015;2(4):423–429. 10.1007/s40572-015-0072-x 26452675

[4] Owusu D , Huang J, Weaver SR, et al. Patterns and trends of dual use of e-cigarettes and cigarettes among US adults, 2015–2018. Prev. Med. Rep 2019;16:101009. 31763161 10.1016/j.pmedr.2019.101009 PMC6861646

[5] El Dib R , Suzumura EA, Akl EA, et al.. Electronic nicotine delivery systems and/or electronic non-nicotine delivery systems for tobacco smoking cessation or reduction: a systematic review and meta-analysis. BMJ Open 2017;7(2):e012680. 10.1136/bmjopen-2016-012680 PMC5337697 28235965

[6] Patil S , Arakeri G, Patil S, et al. Are electronic nicotine delivery systems (ENDs) helping cigarette smokers quit?—Current evidence. J Oral Pathol Med. 2020;49(3):181–189. 31642553 10.1111/jop.12966

[7] Chan GC , Stjepanović D, Lim C, et al. A systematic review of randomized controlled trials and network meta-analysis of e-cigarettes for smoking cessation. Addict. Behav 2021;119:106912. doi:10.1016/j.addbeh.2021.106912 33798919

[8] Hartmann-Boyce, J., McRobbie, H., Butler, AR., et al. Electronic cigarettes for smoking cessation. Cochrane Database of Systematic Reviews 2021 [cited 9 CD010216]; DOI: 10.1002/14651858.CD010216.pub6]. https://www.cochranelibrary.com/cdsr/doi/10.1002/14651858.CD010216.pub6/full. 10.1002/14651858.CD010216.pub4 PMC8094228 33052602

[9] Padon AA , Maloney EK, Cappella JN. Youth-targeted e-cigarette marketing in the US. Tob. Regul. Sci 2017;3(1):95–101. 28083545 10.18001/TRS.3.1.9 PMC5221880

[10] Hoek J , Freeman B. BAT(NZ) draws on cigarette marketing tactics to launch Vype in New Zealand. Tob Control. 2019;28(e2):tobaccocontrol-2019-054967. 10.1136/tobaccocontrol-2019-054967 31315965

[11] Khouja JN , Suddell SF, Peters SE, Taylor AE, Munafò MR. Is e-cigarette use in non-smoking young adults associated with later smoking? A systematic review and meta-analysis. Tob Control. 2021;30:8–15. doi:10.1136/tobaccocontrol-2019-055433. PMC7803902 32156694

[12] Wang TW , Gentzke AS, Neff LJ, et al. Characteristics of e-cigarette use behaviors among US youth, 2020. JAMA 2021;4(6):e2111336–e2111336. 10.1001/jamanetworkopen.2021.11336 PMC8185598 34097049

[13] Greenhalgh, E., Jenkins S, and Scollo M. 18B.3 Prevalence of e-cigarette use. Tobacco in Australia: Facts and issues 2022 28 March 2022]; https://www.tobaccoinaustralia.org.au/chapter-18-harm-reduction/indepth-18b-e-cigarettes/18b-3-extent.

[14] Ministry of Health New Zealand. Annual Update of Key Results 2020/21: New Zealand Health Survey. 2021. https://www.health.govt.nz/publication/annual-update-key-results-2020-21-new-zealand-health-survey. Accessed March 30, 2022

[15] World Health Organisation. Tobacco: E-cigarettes. Q & A Detail 2020 7 April 2021]; https://www.who.int/news-room/q-a-detail/tobacco-e-cigarettes.

[16] Campus, B., Fafard, P., St. Pierre, J., Hoffman, SJ. Comparing the regulation and incentivization of e-cigarettes across 97 countries. Soc. Sci. Med., 2021. 291: p. 114187. 34763132 10.1016/j.socscimed.2021.114187

[17] Smith KE , Ikegwuonu T, Weishaar H, Hilton S. Evidence use in E-cigarettes debates: scientific showdowns in a ‘wild west’of research. BMC Public Health 2021;21(1):1–16. 33593318 10.1186/s12889-021-10396-6 PMC7884966

[18] Mykkänen M , Ikonen P. Media strategies in lobbying process. A literature review on publications in 2000-2018. Academicus ISJ 2019;(20):34–50. doi:10.7336/academicus.2019.20.03

[19] McCausland K , Maycock B, Leaver T, Jancey J. The messages presented in electronic cigarette–related social media promotions and discussion: scoping review. J Med Internet Res. 2019;21(2):e11953. 30720440 10.2196/11953 PMC6379814

[20] van der Tempel J , Noormohamed A, Schwartz R, et al. Vape, quit, tweet? Electronic cigarettes and smoking cessation on Twitter. Int J Public Health. 2016;61(2):249–256. 26841895 10.1007/s00038-016-0791-2

[21] Cole-Lewis H , Pugatch J, Sanders A, et al. Social listening: a content analysis of e-cigarette discussions on Twitter. J Med Internet Res. 2015;17(10):e243. 26508089 10.2196/jmir.4969 PMC4642379

[22] Ayers JW , Leas EC, Allem J-P, et al. Why do people use electronic nicotine delivery systems (electronic cigarettes)? A content analysis of Twitter, 2012–2015. PLoS One. 2017;12(3):e0170702. 28248987 10.1371/journal.pone.0170702 PMC5331961

[23] Kavuluru R , Sabbir A. Toward automated e-cigarette surveillance: spotting e-cigarette proponents on Twitter. J Biomed Inform. 2016;61:19–26.doi:10.1016/j.jbi.2016.03.006 26975599 PMC4893981

[24] McCausland K , Maycock B, Leaver T, et al. E-Cigarette Advocates on Twitter: Content Analysis of Vaping-Related Tweets. J Med Internet Res. 2020;6(4):e17543. 10.2196/17543 PMC7593865 33052130

[25] Lazard AJ , Saffer AJ, Wilcox GB, et al. E-cigarette social media messages: a text mining analysis of marketing and consumer conversations on Twitter. J Med Internet Res. 2016;2(2):e171. 10.2196/publichealth.6551 PMC5187450 27956376

[26] Harris JK , Moreland-Russell S, Choucair B, et al. Tweeting for and against public health policy: response to the Chicago Department of Public Health’s electronic cigarette Twitter campaign. J Med Internet Res. 2014;16(10):e238. 25320863 10.2196/jmir.3622 PMC4210950

[27] Allem J-P , Escobedo P, Chu K-H, et al. Soto, DW., Cruz, TB., Unger, JB. *Campaigns and counter campaigns: reactions on Twitter to e-cigarette education*. Tob Control. 2017;26(2):226–229. 26956467 10.1136/tobaccocontrol-2015-052757 PMC5018457

[28] Leas, EC. , Prochaska, JJ., Ayers, J., Nobles, AL., Henriksen, L. What to do when tobacco advertisers exploit antitobacco social media campaigns to sell tobacco. 2020. 29(2): p. 243. 10.1136/tobaccocontrol-2019-054993 31249102

[29] Consumer Advocates for Smoke-Free Alternatives Association (CASAA). Historical timeline of vaping & electronic cigarettes. 27 April 2021 https://web.archive.org/web/20210408005355/https://casaa.org/education/vaping/historical-timeline-of-electronic-cigarettes/.

[30] Consumer Advocates for Smoke-Free Alternatives Association (CASAA). International Call to Action! Celebrate World Vaping Day by Telling the World Health Organization How E-Cigarettes Have Changed Your Life. 2014 8 April 2021 https://web.archive.org/web/20150116224347/http://blog.casaa.org/2014/09/international-call-to-action-celebrate.html.

[31] Robertson L , Joshi A, Legg T, et al. Exploring the Twitter noise around the Eighth meeting of the Conference of the Parties to the WHO Framework on Tobacco Control. Tob Control. 2022;31(1):50–56. 33177210 10.1136/tobaccocontrol-2020-055889

[32] Tobacco Control Research Group (TCRG). International Network of Nicotine Consumer Organisations (INNCO). Last modified 17 February 2020 23 November 2020. https://tobaccotactics.org/wiki/international-network-of-nicotine-consumer-organisations-innco/.

[33] Tobacco Control Research Group (TCRG). Consumer Choice Center. Last modified 6 February 2020 https://www.tobaccotactics.org/index.php?title=Consumer_Choice_Center. Accessed March 17, 2020

[34] Sollenberger, R. and Bredderman W. Guess Who’s Secretly Backing This “Anti-Smoking” Vape Group. 31 March 2022. https://www.thedailybeast.com/world-vapers-alliance-slams-cigarettes-big-british-american-tobacco-is-secretly-behind-it?ref=scroll.

[35] INNCO. World Vape Day: Testimonials and Statements. 2020 https://web.archive.org/web/20200622013614/https://worldvapeday.org/testimonials/. Accessed May 6, 2021

[36] Bridge G , Flint S, Tench R. A Social Network Analysis of the# SugarTax debate on Twitter. Obesity Abstracts 2019;1. doi:10.1530/obabs.01.OC2.5. PMC10195284 33706816

[37] Hatchard JL , Neto J, Quariguasi F, Vasilakis C, Evans-Reeves KA. Tweeting about public health policy: Social media response to the UK Government’s announcement of a Parliamentary vote on draft standardised packaging regulations. PLoS One. 2019;14(2):1–16. doi:10.1371/journal.pone.0211758 PMC6391026 30807582

[38] Braun, V. and Clarke V. *Thematic analysis, in APA handbooks in psychology* ^ *®* ^. *APA handbook of research methods in psychology, Vol. 2. Research designs: Quantitative, qualitative, neuropsychological, and biological*, H. Cooper, et al., Editors. 2012, American Psychological Association.

[39] Blair, E . A reflexive exploration of two qualitative data coding techniques. J. Methods Meas. Soc. Sci. 2015;6(1):14–29.

[40] McHugh ML. Interrater reliability: the kappa statistic. Biochem Med 2012;22(3):276–282. PMC3900052 23092060

[41] Twitter. Twitter Terms of Service. 2020 https://twitter.com/en/tos. Accessed March 3, 2020.

[42] Colditz JB , Welling J, Smith NA, James AE, Primack BA. World vaping day: Contextualizing vaping culture in online social media using a mixed methods approach. J. Mix. Methods Res. 2019;13(2):196–215.

[43] Consumer Advocates for Smoke-Free Alternatives Association (CASAA). About; Charter; Organizational Goals. 2010. https://web.archive.org/web/20100916181153/http://www.casaa.org/about/charter.asp. Accessed April 27, 2021.

[44] INNCO. World Vape Day: Events. 2020 https://web.archive.org/web/20200623063421/https://worldvapeday.org/events/. Accessed April 1, 2022.

[45] Evans-Reeves, K. and Baker J. Worldwide news and comment. Tob. Control 2022 [cited 10.1136/tobaccocontrol-2022-057288; Available from: https://tobaccocontrol.bmj.com/content/31/2/125.

[46] Ulucanlar S , Fooks GJ, Gilmore A. The policy dystopia model: an interpretive analysis of tobacco industry political activity. PLOS Med.9 2016;13. doi:10.1371/journal.pmed.1002125. PMC5029800 27649386

[47] Dewhirst T. Co-optation of harm reduction by Big Tobacco. Tob Control. 2020. 10.1136/tobaccocontrol-2020-056059 32796080

[48] Peeters S , Gilmore A. Understanding the emergence of the tobacco industry’s use of the term tobacco harm reduction in order to inform public health policy. Tob Control. 2015;24(2):182–189. 24457543 10.1136/tobaccocontrol-2013-051502 PMC4345518

[49] Fenton, E., Robertson, L., and Hoek, J. *Ethics and ENDS*. Tob. Control, 2022. Doi: 10.1136/tobaccocontrol-2021-057078.

[50] Twitter. Ads Content Policies: Tobacco and tobacco accessories. 2022 [cited 31 March 2022 31 March 2022]. https://business.twitter.com/en/help/ads-policies/ads-content-policies/tobacco-and-tobacco-accessories.html.

[51] Lazard AJ , Wilcox GB, Tuttle HM, Glowacki EM, Pikowski J. Public reactions to e-cigarette regulations on Twitter: a text mining analysis. Tob Control. 2017;26(e2):e112–e116. 28341768 10.1136/tobaccocontrol-2016-053295

[52] McCausland K , Maycock B, Leaver T, et al. E-cigarette promotion on twitter in Australia: content analysis of tweets. JPHS 2020;6(4):e15577. 10.2196/15577 PMC7677022 33151159

[53] Hunt D. How food companies use social media to influence policy debates: a framework of Australian ultra-processed food industry Twitter data. Public Health Nutr. 2021;24(10):3124–3135. 33222709 10.1017/S1368980020003353 PMC9884788

[54] Malone RE , Hoek J. Political ineptitude, public anxiety and the undermining of the WHO. Tob Control. 2020;29(4):361–362. 32561575 10.1136/tobaccocontrol-2020-055978

[55] Freeman, B, Watts C, Astuti, PAS. Global tobacco advertising, promotion and sponsorship regulation: what’s old, what’s new and where to next? Tob Control. 2022;31(2):216–221. 35241591 10.1136/tobaccocontrol-2021-056551

