# Supplementary material for: An Analysis of Arguments Advanced via Twitter in an Advocacy Campaign to Promote Electronic Nicotine Delivery Systems
Source: Nicotine Tob Res. 2022 Oct 21;25(3):533–40. doi: 10.1093/ntr/ntac237 (PMC9910155; doi:10.1093/ntr/ntac237)
Supplement: ntac237_suppl_Supplementary_Material_S1 [file ntac237_suppl_supplementary_material_s1.docx]

| **Theme & sub-theme** | **Supporting Quotations^[[1]](#footnote-2)^** |
| --- | --- |
| **HARM REDUCTION** |  |
| *Harm reduction* | Happy world vape day people!! Sounds like a good day to switch from smoking to a healthier alternative!! #WorldVapeDay #THR #vapenotsmoke #vapelife  #WorldVapeDay is a celebration of our choice to make the switch to a healthier, smoke-free lifestyle. We seek to raise awareness about the significant health, social, and economic benefits of e-cigarettes/vapor products for people who smoke.  One day after #WorldVapeDay we now celebrate #WNTD2020. What does this mean to you? To me, it means there is hope one day all smokers will quit smoking- be it by #vaping #snus #HnB or whichever method works for the individual, it’s all harm reduction! #SayYestoTHR #ExposeTobacco |
| *At least 95% less harmful* | #WorldVapeDay #DiaMundialSinTabaco2020 #WorldNoTobaccoDay #TobaccoExposed The lifetime cancer risk of vaping has been assessed to be under 0.5% of the risk of smoking. There is negligible risk to others from second-hand e-cigarette vapour.  We already know #vaping is at least 95% safer than smoking! #WorldVapeDay #WorldTobaccoDay #WorldNoTobaccoDay2020  If you are not promoting 95% safer flavored #ecigs to ALL SMOKERS, YOU ARE PROMOTING SMOKING! #ABILLIONLIVES #Vaping #WorldVapeDay #WorldTobaccoDay #WorldNoTobaccoDay |
| *Nicotine not harmful* | Tobacco harm reduction (THR) is a harm reduction strategy aimed at reducing illness, injury and death in people who use nicotine. Most people who use nicotine smoke it, and smoke, not nicotine, is responsible for the vast majority of tobacco related harm. #WorldVapeDay  Nicotine does NOT cause cancer. Smoke and tar does. Vaping does not have smoke. It is simple to understand why it is safer than smoking. Why ban it then?  Why are we to give up nicotine consumption when nicotine itself is no more harmful than caffeine? #WorldVapeDay #SayYesToTHR |
| *Improved health outcomes* | Don't let any misinformed "expert" or government body getting rich off of tobacco money try to dissuade you from finding a safer alternative to smoking combustible cigarettes that kill 480k a year in the US. Switch to vaping and improve your heart and lung health. #WorldVapeDay  A study from the University of Dundee, published in November 2019 and funded by the British Heart Foundation, suggests that vaping may be less harmful to your blood vessels than smoking cigarettes.  #WorldVapeDay #SayYesToTHR #VapingSavesLives  #WorldVapeDay #DiaMundialSinTabaco2020 #WorldNoTobaccoDay #TobaccoExposed There's more evidence than ever that e-cigarettes are safer than smoking, and a way to give up altogether Swapping cigarettes for an ecig can improve your symptoms of lung conditions like asthma and COPD |
| *Vaping is not smoking* | Drinking water from a glass looks exactly the same as drinking vodka. No one denies that the second one has a much higher risk on health deterioration. Same goes for smoking and #vaping , both look similar, but the latter bears much less risk. #worldvapeday #WorldNoTobaccoDay  Vaping is NOT smoking. It helped me quit tobacco. 10 crore smokers in India can also benefit if the vape ban is lifted. #WorldVapeDay #EndVapeBan #WNTD2020  They can say that vapers are "still smoking". That doesn't change the facts. Something must at least contain smoke to be rightfully called smoking. Where there's no smoke, there's no one smoking. Shouldn't be to hard to grasp, @WHO.#WorldVapeDay #SayYesToTHR #TobaccoExposed |
| *Second-hand vape not harmful* | The evidence is clear that second hand smoke exposure is harmful. To date, there are no proven health risks of second hand vaping to the health of bystanders. Vaping could prevent millions of deaths from second-hand smoke. #WNTD2020 #TobaccoExposed #WorldVapeDay  E-liquid is composed of nicotine, propylene glycol and/or glycerine & flavourings. Unlike cigarettes, no side-stream aerosol is emitted by e-cigarettes into the atmosphere, just the exhaled vapor. #WNTD2020 #TobaccoExposed #WorldVapeDay  Just me again on #WorldVapeDay reminding you, under realistic conditions, new-generation vapor products emit minimal aldehydes/g liquid at both low and high power. You know, science!  #SayYesToTHR #ExposeTobacco |

| **SMOKING CESSATION** |  |
| --- | --- |
| *Testimonial* | Im [sic] 49. I switched to vaping 7 years ago. Started at 18mg. Now enjoying 3mg and staying with it thanks to all the great flavors. I trust the unbiased science and my own improved health. I fully support THR, it works. #WorldVapeDay #tobaccofree #VapingSavesLives  I am 34 years old and flavored vapor products helped me quit a 15 year habit. Adults like flavors! #grimmarmy #WorldVapeDay #nowaypmta #TobaccoExposed #vapingsavedmylife #vaping  I smoked for 35yrs, until some1 [sic] introduced me to #vaping. Almost 6 years ago (5 days shy-6yrs) I #quitsmoking at NO cost to taxpayer! I'm in my 50's. I vape lots of #flavors tastebuds don't die at 20! #adultsloveflavors If you struggle with smoking #SayYesToTHR #WorldVapeDay |
| *Improved symptoms* | I'm 56, smoked for over 30 years, finally quit by vaping, and the flavors are what made it work. Almost 5 years smoke free, and feeling and breathing a lot better than before. #WorldVapeDay  After smoking for 40 years I started vaping approx 7 years ago. All of my "smoking" family went to vaping that year. No more bouts of bronchitis, pneumonia, etc. We all feel great. And we are grateful! #WorldVapeDay #vapingsaveslives  Thanks to #vaping I haven't touched Cigarette [sic] for almost a year now. Tons of health complications have gone automatically as I stayed away from the cancer stick. No, I have no business related to vaping.  #WorldVapeDay #EndVapeBan #WNTD2020 |
| *ENDS save lives* | Vaping helps save lives... Tweeting this as I finish my back workout. Still on this health journey, thanks to vaping, the vaping community and vape companies! #WorldVapeDay  Thanks to vaping I’m still around. I’m going to be able to see the birth of my first child. Vaping saved my life. Blue Slushie flavored Vapor kept me off deadly combustible cigarettes. I owe my life to vaping. #WorldVapeDay #WeVapeWeVote #SayYesToTHR  95% safer. saves lives. #TobaccoExposed #Alabamavapemob #SayYesToTHR #worldvapeday #WTHRD #WHO #WorldNoTobaccoDay |

| *ENDS support quitting* | According to Public Health England e-cigarettes could help more people quit smoking 🚭#SayYesToThr #WorldVapeDay  Vaping is 95% safer than smoking, yes some of us werent [sic] able to give up smoking but vaping helped us to give up smoking and its 4000 chemicals and tar for a healthier alternative Vaping saves lives! #EndVapeBan #notobaccoday #WorldVapeDay  This is one of the fantastic people I know who is drop dead serious about #WorldVapeDay and #SayYesToTHR [sic] via vapor products to stop smoking! |
| --- | --- |
| *Effects of smoking* | Unlike patches, pills & hotlines, vaping is an existential threat to smoking. The war on vaping helps Big Tobacco &amp; protects a status quo where 8M people die from smoking each year. Anti-vaping crusades are pro-death. #WorldVapeDay #WNTD2020 #TobaccoExposed  A billion smokers in the world, half of whom will die prematurely from tobacco related illnesses. Despite clear evidence to show vaping is safer, nations play politics with human lives. Why is @FCTCofficial supporting this? #WorldVapeDay #EndVapeBan #TobaccoExposed #WNTD2020  We are survivors. That's what vaping did for us. 8 million annual deaths due to smoking. Thankfully we are not among the 8 millions because vaping saved our lives. @WHO, @WHOEurope, @CDCgov, @BloombergDotOrg would rather see us dead than vaping. #WorldVapeDay #SayYesToTHR |
| *ENDS’ superiority* | Vaping is the most effective aid to quitting smoking ever known. Smoking is the worlds' greatest cause of unnecessary death and disease, taking 78,000 lives every year in the UK alone, and responsible for half million UK hospital admissions in the same period. #WorldVapeDay  E-cigarettes are nearly twice as effective for smoking cessation than nicotine-replacement therapy, when both products were accompanied by behavioral support according to a rigorous randomized control trial #WorldVapeDay  Vaping is much less harmful than smoking, and much more effective in smoking cessation than nicotine gum and patches. #WorldVapeDay #SayYesToTHR #TobaccoExposed |

| *Cessation success* | @CDCTobaccoFree The American people have a right to know the truth about flavored nicotine e-liquid vaping; 0 deaths 18% cessation, 80% smoker transition, 》95% less harmful. #WorldVapeDay  @AmericanCancer You are a true cancer to society. We have T21 nationwide just like alcohol / cigarettes. Flavored nicotine e-liquid vaping has never killed anyone and has an amazing 18% cessation success rate. Bans/taxes just force adults/youth back to smoking that kills 1,300/day #WorldVapeDay  “The overall population cessation rate for 2014-14 was significantly higher than that for 2010-11, 5.6% v 4.5% and higher than those for all other survey years.” #WorldVapeDay |
| --- | --- |
| **RIGHTS AND JUSTICE** |  |
| *Access to flavours* | *Quitting smoking was made possible for me and millions on others by way of flavored vapes. Today I celebrate #WorldVapeDay chucking some BlackBerry Jam monster clouds on my front porch, as my wife, sadly puffs on a menthol cig. She'll figure it out someday...*  *I love gummy candy, anything grape, rootbeer [sic]. "I" at 58 vape flavors. Please stop, stop this ban on flavors. #WorldVapeDay #SayYesToTHR #TobaccoExposed My word, we ARE adults who like flavors and quit smoking by vaping flavors. Almost 4 years smoke free*  *I feel I should point out the Elephant in the room, Nicotine products are ALREADY age restricted, by law youngsters cannot buy them, rather than the flavours, the real target should be those breaking that law....#SayYesToTHR #WorldVapeDay* |
| *Right / freedom to choose* | The war against smoking has turned into a war against nicotine. Why is nicotine in patches, gums, and in oral and nasal sprays totally accepted and is vilified when in ecigs [sic]? Trampling on Human Rights is unjustifiable! #WorldVapeDay #SayYesToTHR #WNTD2020 #TobaccoExposed  Proud & inspired by everyone this weekend sharing their stories & fighting for their right to #TobaccoHarmReduction. But remember, we're not done. Do not slow down. Keep the pressure on. #HarmReduction is a HUMAN RIGHT. #WorldVapeDay #SayYesToTHR #RiseAndVape #WeVapeWeVote  I could not have said it any better!!! It’s my human right!! Vaping is a safer alternative to smoking combustible cigarettes and it’s your right too! Fight for your right! #WorldVapeDay #SayYesToTHR #ExposeTobacco |
| *Vaping is for adult smokers* | This is mind blowing why the @WHO would target a 95% safer alternative to 🚬. To stand on that side of the line & use children to try stop ADULTS from quitting smoking..i dont [sic] comprehend. I'm thankful for the opportunity to vape. #WorldVapeDay #SayYesToTHR #flavorssavelives  It was already banned for minors! This is legislation is meant to deny ADULTS from smoking cessation products. Hopefully it dies in the Senate. #WeVapeWeVote #WorldVapeDay dictator.  #WorldVapeDay #Flavours are key for adults to becoming #SmokeFree with #Vape. Cigarettes only came in tobacco and menthol when we starting #smoking, #Flavours saved us. #FactsNotFiction |
| *ENDS industry consumer-driven* | Vaping is a grass-roots, consumer-driven solution to smoking. Consumers embraced them and were the drivers behind improved technology, safety and flavourings. The Tobacco Industry was late to the game. #WorldVapeDay  Smokers found a solution to quitting smoking and many started small businesses that provide safer alternatives to people who smoke. Vaping is a grass-roots, consumer-driven solution to smoking. #WorldVapeDay  #WorldVapeDay is quickly becoming my favourite day of the year. Nothing but love for Vapers and THEIR industry. Grassroots and real, with only care for all the smokers still out there accessing far safer alternatives. Thank you all. ☮&amp;💜💜💜 #SayYesToTHR |
| *Involve consumers* | We are consumers. We do not speak for the tobacco industry or share their interests. We are not their agents. We advocate for vaping because it saved our lives. We advocate for vaping because we KNOW it works. Listen to consumers. #WorldVapeDay  Vaping just works. We are all proof of that. #WorldVapeDay #WorldNoTobaccoDay  Consumers are the stakeholders most profoundly impacted by @WHO and @FCTCofficial tobacco policies. Please hear us as we ask you to #SayYesToTHR on #WNTD2020 and #WorldVapeDay |
| *Don’t deserve to die* | Cigarettes kill half of their users. Nicotine dependence shouldn’t be a death sentence. Vaping saves lives. #WorldVapeDay  Your false quest to "protect kids" is keeping adults like Jeannie from getting the products she uses to stay off cigarettes. Stop punishing adults, they deserve to live long and healthy [sic] |
| *Refutes ‘gateway’ hypothesis* | The ones that say no to THR are the ones profiting and collecting blood money from smokers. They lie about vaping so that you will hate the idea too. Truth is that vaping leads to not smoking. #WorldVapeDay  Concerns about youth vaping are understandable but the novelty of e-cigarettes can make us forgetful of the fact that nicotine and tobacco use is endemic among youth & involves extremely hazardous products before vaping. Youth smoking rates are rapidly declining. #WorldVapeDay  Hey there @MassGovernor your vape ban fixed youth Vaping! The teenager next door who used to vape a juul, now smokes cigarettes! You must be so proud to start a new generation of smokers! Save Vapor Products. #WorldVapeDay #flavors #ivapeivote |
| *Youth ENDS trial inevitable* | On #WorldVapeDay we celebrate that tens of millions of smokers have found a safer alternative and escaped their addiction to smoking. Vaping is also less addictive than smoking, even for kids who will always experiment with "forbidden fruit."  Young people shouldn’t use any nicotine, but the reality is that some of them do. We must acknowledge this reality & make sure vaping prevention education programs don’t mislead youth who are going to use nicotine despite our efforts to stop them. #WNTD2020 #WorldVapeDay  I have warned that anti-vaping campaigns are turning e-cigs into a "forbidden fruit" for curious teens. Luckily, youth experimentation is much different than addiction but anti-vape movements are counterproductive. On #WorldVapeDay please remember, for smokers #VapingSavesLives |
| *Right to know the truth* | the public needs to know the truth about #vaping please read and realize that we fight for this technology because #VapingSavesLives we will not go back to cigarettes #WorldVapeDay #SayYesToTHR #TobaccoExposed  Equating e-cigs with cigarettes ignores consensus based on high quality studies showing substantial differences in the risks associated with smoking and vaping. The ethical duty is to examine all the research including the+. Discarding the good is immoral.  It is my #Honor and #privilege to give #promotion to #worldvapeday and to #SayYesToTHR because #vapingsaveslives and #world #Government #Leaders #need the #TruthBeTold and should #Listen |

| **OPPOSITION TO ENDS RESTRICTIONS** | |
| --- | --- |
| *Criticism of those perceived as opposing ENDS* | For #WorldNoTobaccoDay an international group of independent experts with no conflicting links to industry has sharply criticized the WHO for its backward-looking approach to innovation and new technology, such as vaping products. #WorldVapeDay  This, right here, is why #vaping has gotten the reputation it does- not because of actions the industry has taken, but from “Public Health” orgs [sic] that profit from people smoking. Vaping is harm reduction. #WorldVapeDay #SayYestoTHR #vapingsaveslives  Everyone knows @WHO failed in tackling Covid-19 and lied for its funder China. It is also lying on vaping for big funder Bloomberg. Lives of 1 billion smokers should come first. |
| *Misinformation about ENDS* | Never smokers are not being attracted to #vaping so don't let misinformation by anti-harm lobbyists cloud your judgement for best public health. #ANTZLied #WorldVapeDay  Nicotine without smoke is not very harmful, but disinformation about the relative risk of nicotine kills. #WorldVapeDay  I completely agree with you. @PMOIndia are you reading this? Your ban is not based on science but on unfounded theories #EndVapeBan #WorldVapeDay #WNTD2020 |
| *Repeal ENDS ban* | I had quit smoking with the help of vaping before the ban.the ban on purchasing and transport restrictions on e cigs [sic] pushed me back to smoking . Please remove the ban. #WorldVapeDay #EndVapeBan #WNTD2020  On this #worldVapeDay, got to tell the world. Vaping, it saved my life, its [sic] the best NRT. We have to #EndVapeBan in India #NoTobaccoDay  HIV #HarmReduction: condoms ✓ Car accident HR: security belt ✓ Motorbike accident HR: helmet ✓ Tobacco HR: vape -& BANNED WHY? #WorldVapeDay #EndVapeBan #TobaccoExposed #WNTD2020 |
| *Restrictions unjustified or unrealistic* | Despite the known benefits of vaping, prohibitions on safer nicotine products are rising, including in countries where the number of people who smoke is predicted to increase due to population growth. #WorldVapeDay  These are adult only products #WorldVapeDay Proven: prohibition does not work What else should we ban, movies? comic books?  The number of people directly affected by the serious and real harms of combustible tobacco is vast. Eradicating nicotine use is an unrealistic goal, but eradicating the risk of combustible tobacco is not. |
| *Unintended consequences* | "Several countries which banned e-cigarettes including Mexico, Brazil and Thailand saw a booming black-market - making it difficult for government to regulate the sales of these products"  The history of anti-drug campaigns is replete with some spectacular failures, like the US/Canada DARE (drug awareness education & research) campaign, which has actually been linked to *increased* drug use among youth. #WorldVapeDay  Proposing that we actually increase health inequality between the developed and developing world by denying people who can least access healthcare the means to prevent harm is lunacy. #WorldVapeDay #TobaccoExposed #WNTD2020 |
| *International comparisons* | The UK is a great example of a country encouraging smokers to switch. The switching rate is much higher than in other countries because they have embraced it. #WorldvapeDay  In the United States and Canada, authoritative medical science bodies and government health agencies acknowledge the low risk of vaping relative to smoking & the potential health benefits of switching from smoking to vaping. #WorldVapeDay  A problem with #vaping policy in Luxembourg and many countries is educating policymakers. Many of them see smoke and instantly equate it to smoking cigarettes. #WorldVapeDay |
| **EXAMPLES OF NO ARGUMENT/ EXCLUDED TWEETS** | |
|  | Today is #WorldVapeDay  Not just for #WorldVapeDay  #WorldVapeDay This makes so much sense @GregHuntMP @TGAgovau @CancerCouncilOz  #worldvapeday is happening everywhere!  This is #worldvapeday but I am taking a moment of sadness and reflection as violence has gripped #America. These #riots2020 are senseless as well as the lives being lost. No good can come from this.  I SUPPORT #WorldVapeDay #NoTobaccoDay #EndVapeBan  #WorldVapeDay this Saturday  First celebrated in 2012, World Vape Day has been held with varying levels of community engagement on various dates. This year, the celebration has a permanent home on our calendars: May 30th, one day before WHO’s annual “World No Tobacco Day” (May 31). #WorldVapeDay #WNTD2020  #WorldVapeDay Millions of e-cigarette users around the world will celebrate 'World Vape Day' on May 30  #worldvapeday save vaping @realDonaldTrump  Give a great eliquid company a try!! #WorldVapeDay #SayYesToTHR  **C**𝐡𝐨𝐨𝐬𝐞 𝐖𝐢𝐬𝐞𝐥𝐲! There are approximately 600 ingredients in both Food and cigarettes. #WorldVapeDay #WorldNoTobaccoDay #TobaccoExposed #WNTD2020 #NoTobacco #notobaccoday #sundayvibes #QuitSmoking #NotGoodForHealth #DontStart |

1. Most ‘mentions’ of other Twitter accounts, and any URLs embedded in a tweet have been removed for privacy reasons. [↑](#footnote-ref-2)
