# Supplementary material for: An Analysis of Arguments Advanced via Twitter in an Advocacy Campaign to Promote Electronic Nicotine Delivery Systems
Source: Nicotine Tob Res. 2022 Oct 21;25(3):533–40. doi: 10.1093/ntr/ntac237 (PMC9910155; doi:10.1093/ntr/ntac237)
Supplement: ntac237_suppl_Supplementary_Material_S2 [file ntac237_suppl_supplementary_material_s2.docx]

**Supplementary File 2. Stages undertaken in data collection and analysis**

**#WorldVapeDay**

**58,551 tweets collected**

**4,387 original tweets**

**N=610 coded as ‘no argument’**

**Primary analysis**

Qualitative thematic analysis of tweets

(n=1,590 tweets coded as an argument, position and/ or call to action)

**Excluded tweets:**

**Non-English language**

**Retweets**

**Supplementary analysis**

Content analysis of most frequent arguments

**Supplementary analysis**

Descriptive analysis of most common hashtags

**Random sample of n=2,200 tweets drawn**

**39,471 tweets**
