# Supplementary material for: An Analysis of Arguments Advanced via Twitter in an Advocacy Campaign to Promote Electronic Nicotine Delivery Systems
Source: Nicotine Tob Res. 2022 Oct 21;25(3):533–40. doi: 10.1093/ntr/ntac237 (PMC9910155; doi:10.1093/ntr/ntac237)
Supplement: ntac237_suppl_Supplementary_Material_S3 [file ntac237_suppl_supplementary_material_s3.docx]

**Supplementary File 3. Definition and frequency of non-prevalent arguments about ENDS, tweets categorised as a ‘Call to Action’, and excluded tweets**

| **THEME;** *Argument* *or Action* | **Definition** | **Frequency*** |  |
| --- | --- | --- | --- |
| **HARM REDUCTION** |  |  |  |
| *Can’t evade all risk in life* | Argues nothing is without risk (e.g. drinking caffeine/ alcohol use), implying some risk associated with nicotine is acceptable. | 6 |  |
| **SMOKING CESSATION** |  |  |  |
| *Prevent relapse* | Argues or suggests ENDS could help people stay smoke-free and prevent relapse. | 5 |  |
| *Make cigarettes obsolete* | Claims or alludes to idea that ENDS could make combustible cigarettes obsolete. | 5 |  |
| *Dual use* | Makes any reference to dual use of ENDS with cigarettes. | 2 |  |
| **OPPOSITION TO ENDS RESTRICTIONS** | | |  |
| *Criticism of ENDS or tobacco industry* | Expresses criticism or mistrust of tobacco companies or ENDS retailers or manufacturers. | 9 |  |
| **COMMERCIAL/ ECONOMIC** |  |  |  |
| *Promotions or marketing* | Promotes particular ENDS products or brands, with or without special offers/ discounts. | 7 |  |
| *Boost economy* | Refers to the idea that liberal ENDS policies will help the economy. | 5 |  |
| *Job losses* | Refers to the idea that ENDS restrictions will negatively impact ENDS retailers/ manufacturers, resulting in job losses. | 5 |  |
| **OTHER** | | |  |
| *Unsupportive of ENDs* | Tweets does not appear to support ENDS. | 1 |  |
| **CALL TO ACTION** | | |  |
| *Call to Action* | Encourages people to perform an advocacy activity or awareness raising e.g. “Share your story about going vape-free!”. | 147 |  |
| *General* | Tweet encourages some sort of action but is not specific e.g. "get Involved" or "let's celebrate and educate". | *82* |  |
| *Share stories, photos* | Encourages people to share their stories or images about ENDS to social media or generally. | *42* |  |
| *Join a group* | Encourages people to join a group in some sort of advocacy action e.g. "join CASAA in fighting for harm reduction". | *11* |  |
| *Answer a survey or a question* | Encourages people to take part in a survey, answer a question with a retweet or similar. | *4* |  |
| *Attend an event* | Encourages attendance at an event; could be a physical or an online event e.g. webinar, launch of report etc. | *4* |  |
| *Send a message to politicians* | Tweet appears to be encouraging people to send a message to regulators/ government officials/ policy-makers. | *4* |  |
| **NO ARGUMENT (EXCLUDED)** |  |  |  |
| Tweet does not put forward an argument, position, or call to action. May not contain sufficient text to code. | | 610 |  |
